# Fixed Dose Combinations as an Advantage for the Treatment of Pediatric Tuberculosis: A Narrative Review

**DOI:** 10.3390/ph19060806

**Published:** 2026-05-22

**Authors:** Susanna Esposito, Beatrice Rita Campana, Gaia Giorgia Arnesano, Nicola Principi

**Affiliations:** 1 Pediatric Clinic, Department of Medicine and Surgery, University Hospital of Parma, 43126 Parma, Italy; beatricerita.campana@unipr.it (B.R.C.); gaiagiorgia.arnesano@unipr.it (G.G.A.); 2 Università degli Studi di Milano, 20122 Milan, Italy

**Keywords:** pediatric tuberculosis, fixed-dose combinations, adherence, pharmacokinetics, WHO guidelines, child-friendly formulations

## Abstract

**Background:** Pediatric tuberculosis (TB) remains a major global health concern, accounting for a substantial proportion of TB-related morbidity and mortality worldwide. Treatment in children is particularly challenging due to age-specific pharmacokinetics, difficulties in drug administration, poor palatability, and reliance on caregivers for adherence. **Objectives:** This narrative review aims to evaluate the advantages and limitations of fixed-dose combinations (FDCs) in the treatment of pediatric TB, with a focus on adherence, pharmacological considerations, clinical outcomes, and implementation challenges. **Methods:** A narrative review of the literature was conducted, including clinical studies, pharmacokinetic analyses, programmatic data, and international guidelines related to the use of FDCs in pediatric TB management. **Results:** Evidence indicates that pediatric FDCs significantly improve treatment adherence by reducing pill burden and simplifying dosing regimens. They also decrease the risk of medication errors and inadvertent monotherapy, thereby contributing to the prevention of drug resistance. The availability of dispersible, child-friendly formulations has enhanced acceptability and ease of administration. However, limitations persist, including reduced flexibility in dose individualization, challenges in identifying the causative agent in adverse drug reactions, and variable access across settings. Pharmacokinetic concerns, particularly regarding rifampicin exposure, have been addressed in newer WHO-recommended formulations. **Conclusions:** FDCs represent a critical advancement in pediatric TB management and are strongly supported by international guidelines. Further research is needed to optimize formulations, ensure equitable access, and evaluate long-term clinical outcomes in diverse pediatric populations.

## 1. Introduction

Tuberculosis (TB) continues to be one of the leading infectious causes of death globally, with children representing a particularly vulnerable population [1,2,3,4,5]. Despite significant advances in diagnosis and treatment, TB remains a major public health challenge, especially in low- and middle-income countries where healthcare resources are limited and the burden of disease is highest [1,2]. It is estimated that over one million children develop TB each year, with a substantial proportion remaining undiagnosed, untreated, or unreported due to diagnostic limitations and gaps in surveillance systems [1,6,7,8]. Childhood TB is often under-recognized, contributing to preventable morbidity and mortality [8,9,10].

Pediatric TB differs significantly from adult disease in terms of clinical presentation, disease progression, and response to therapy [11,12,13,14,15]. Children are more likely to develop severe forms of TB, such as miliary TB and TB meningitis, particularly in younger age groups and in those with compromised immune systems [11,13,15]. Moreover, pediatric TB is typically paucibacillary, meaning that bacterial loads are low, which makes microbiological confirmation challenging [12,14,16,17]. As a result, diagnosis often relies on a combination of clinical, radiological, and epidemiological criteria, increasing the risk of both underdiagnosis and overtreatment [12,14].

The management of pediatric TB presents unique pharmacological and practical challenges [2,3,18,19,20,21]. Age-related changes in drug absorption, distribution, metabolism, and excretion can significantly influence drug exposure, necessitating careful dose adjustments based on weight and developmental stage [22,23,24,25,26,27]. In addition, the lack of child-friendly formulations has historically complicated treatment delivery [2,3,19]. Many anti-TB drugs were originally developed for adults and required manipulation, such as crushing tablets or preparing suspensions, which could compromise dosing accuracy and drug stability [19,20].

Adherence to treatment is another critical issue in pediatric TB care [2,18,20,21]. Standard TB regimens are prolonged, typically lasting six months or longer, and require the consistent administration of multiple drugs [2,18,28]. In children, adherence is heavily dependent on caregivers, who may face challenges related to health literacy, socioeconomic constraints, and access to healthcare services [13,20]. Poor adherence can lead to treatment failure, relapse, and the development of drug-resistant TB, which poses a significant threat to global TB control efforts [6,7,9,29].

Historically, treatment regimens required the administration of multiple drugs as separate formulations, often with differing dosing schedules and instructions [2,3,19]. This complexity increased the likelihood of medication errors, including incorrect dosing or omission of one or more drugs, ultimately compromising treatment effectiveness [19,20]. Furthermore, issues such as poor palatability and difficulty swallowing tablets further reduced acceptability among children, negatively impacting adherence [19,20].

In this context, fixed-dose combinations (FDCs) have emerged as a key strategy to simplify TB treatment [3,18,29]. FDCs combine two or more first-line anti-TB drugs—such as isoniazid, rifampicin, pyrazinamide, and ethambutol—into a single dosage form [18,19,29]. This approach reduces pill burden, simplifies dosing, and helps ensure that all essential drugs are taken simultaneously, thereby minimizing the risk of functional monotherapy and the development of drug resistance [18,29,30].

In recent years, the development of pediatric-specific FDCs, including dispersible and palatable formulations, has further enhanced their utility [18,29]. These formulations are designed to align with WHO-recommended weight bands, allowing for more accurate dosing and improved ease of administration [18,26,29]. As a result, FDCs are increasingly recognized as a cornerstone of modern pediatric TB management [18,20,29]. This review aims to explore their role in improving treatment outcomes, while also addressing remaining challenges and areas for future research. The novelty of this narrative review lies in its focused and updated evaluation of pediatric FDCs as a key component of contemporary TB management. Unlike previous reviews that have addressed pediatric TB treatment more broadly, this article specifically integrates recent WHO recommendations, the introduction of child-friendly dispersible formulations, and emerging pharmacokinetic evidence regarding first-line anti-TB drugs in children. Particular attention is given to how newer pediatric FDCs have addressed historical concerns related to dosing accuracy, palatability, adherence, and rifampicin exposure. By combining clinical, pharmacological, and programmatic perspectives, this review provides an updated synthesis of the advantages and remaining limitations of FDCs, while identifying unresolved research needs related to dose optimization, special pediatric populations, and equitable global access.

## 2. Methods

This narrative review was conducted to synthesize current evidence on the role of FDCs in the treatment of pediatric TB. A comprehensive, non-systematic literature search was performed using PubMed/MEDLINE, Scopus, and Web of Science. In addition, relevant documents and guidelines from international organizations, particularly the WHO, were manually searched to ensure inclusion of key policy and technical reports.

The literature search covered publications from January 2010 to March 2026, in order to capture the period during which pediatric dosing recommendations, child-friendly formulations, and WHO-endorsed FDC strategies have substantially evolved. Earlier landmark studies were also considered when they provided essential background information on pediatric TB pharmacology, drug dosing, or formulation-related challenges.

Search terms included combinations of keywords and Medical Subject Headings (MeSH) such as “pediatric tuberculosis”, “childhood TB”, “fixed-dose combinations”, “anti-tuberculosis drugs”, “adherence”, “pharmacokinetics”, “child-friendly formulations”, and “WHO guidelines”. Boolean operators, including AND and OR, were used to refine the search strategy. The search was limited to articles published in English.

Eligible sources included randomized controlled trials, observational studies, pharmacokinetic analyses, systematic reviews, narrative reviews, programmatic reports, and international guidelines focusing on pediatric populations or providing formulation-related insights applicable to children. Adult studies were considered only when they provided relevant pharmacological or formulation-related information that could inform pediatric FDC use. Studies focusing exclusively on multidrug-resistant TB regimens or non-standard therapies were excluded unless they contributed meaningful insights into FDC use.

After screening titles, abstracts, and full texts for relevance, 32 sources were included in the final synthesis, comprising clinical studies, pharmacokinetic investigations, programmatic reports, reviews, and WHO guidance documents. Data were extracted and synthesized qualitatively, with emphasis on predefined thematic areas: pharmacological rationale, adherence and treatment outcomes, pharmacokinetics and drug exposure, safety and tolerability, child-friendly formulations, implementation challenges, and evidence gaps. Given the narrative nature of the review, no formal risk-of-bias assessment or meta-analysis was performed; however, greater weight was assigned to WHO recommendations, multicenter studies, pharmacokinetic analyses, and high-quality clinical evidence when available.

## 3. Pharmacological Rationale for Fixed-Dose Combinations

FDCs are designed to deliver the core first-line anti-tuberculosis agents—isoniazid, rifampicin, pyrazinamide, and ethambutol—in fixed proportions within a single dosage form [18,19,29]. The pharmacological rationale underlying this approach is rooted in both microbiological and therapeutic principles [19,30]. TB treatment requires the simultaneous use of multiple drugs with different mechanisms of action to effectively eliminate *Mycobacterium tuberculosis* populations, including actively replicating bacilli and dormant subpopulations [19,30]. The combination of drugs reduces bacterial load more rapidly and prevents the emergence of resistant strains [6,7,30].

From a pharmacodynamic perspective, the use of multiple agents targets different metabolic pathways within the *Mycobacterium* [19,30]. Isoniazid inhibits mycolic acid synthesis, rifampicin blocks RNA polymerase activity, pyrazinamide disrupts membrane energetics under acidic conditions, and ethambutol interferes with cell wall arabinogalactan synthesis [19,30]. The concurrent administration of these agents produces a synergistic or additive bactericidal effect, which is essential for shortening treatment duration and improving cure rates [19,28,30].

FDCs ensure that these drugs are administered together in fixed ratios, thereby minimizing the risk of selective drug intake [18,29]. This is particularly important in pediatric settings, where reliance on caregivers increases the potential for dosing errors or omission of individual drugs when given separately [20,29]. By guaranteeing simultaneous drug exposure, FDCs help maintain effective plasma concentrations of all components, reducing the risk of functional monotherapy and the subsequent development of drug resistance [29,30].

Pharmacokinetic considerations are especially relevant in children [22,23,24,25,26,27]. Developmental changes in hepatic enzyme activity, gastric pH, intestinal motility, and renal clearance can significantly alter drug absorption, distribution, metabolism, and excretion [22,23,24,25]. For example, rifampicin metabolism is influenced by age-dependent enzyme maturation, while isoniazid clearance varies according to acetylator status [22,23,24,25]. These factors necessitate weight-based dosing strategies and careful formulation design to ensure adequate drug exposure across different pediatric age groups [18,25,26,27].

The pharmacokinetic and pharmacodynamic profile of first-line anti-TB drugs in children is influenced by substantial interindividual variability related to age, body weight, nutritional status, developmental physiology, comorbidities, and genetic background [22,23,24,25,26,27]. Compared with adults, young children often have lower and more variable plasma concentrations of key drugs, particularly rifampicin and isoniazid, because of differences in gastric pH, intestinal transit time, hepatic enzyme maturation, body water and fat composition, plasma protein binding, and renal clearance [22,23,24,25]. Rifampicin exposure, usually assessed by peak concentration and area under the concentration–time curve (AUC), is especially relevant because suboptimal concentrations may reduce bactericidal activity and contribute to delayed microbiological response [23,24,27]. Isoniazid exposure is additionally affected by *NAT2* acetylator status, with rapid acetylators at greater risk of lower concentrations and slow acetylators at greater risk of toxicity [22,25]. From a pharmacodynamic perspective, rifampicin efficacy is mainly associated with exposure-dependent activity, commonly reflected by the AUC/minimum inhibitory concentration (MIC) ratio, whereas isoniazid shows early bactericidal activity that depends on achieving adequate peak concentrations [23,24,25,30]. Weight-band-based pediatric FDCs were developed to improve dose standardization and increase the likelihood of achieving target exposures [18,26,29]; however, fixed ratios cannot fully account for variability in maturation, nutritional status, HIV co-infection, drug–drug interactions, or pharmacogenetic differences [25,27,31]. Therefore, although modern pediatric FDCs have improved the pharmacokinetic performance of first-line therapy, quantitative exposure remains heterogeneous, and further population pharmacokinetic studies, therapeutic drug monitoring where feasible, and pharmacodynamic target-based dose optimization are needed, particularly in infants, malnourished children, children living with HIV, and those with severe or extrapulmonary TB.

Modern pediatric FDCs are formulated according to WHO-recommended weight bands, which aim to standardize dosing while accommodating physiological variability (Table 1) [15,16].

These formulations are often dispersible, allowing tablets to be dissolved in water to facilitate administration [18,29]. This approach not only improves ease of use but may also enhance drug bioavailability compared to crushed adult tablets, which were historically used off-label in children [19,29].

Another important pharmacological aspect of FDCs is the potential to improve bioavailability through optimized formulation [23,24,29]. Advances in pharmaceutical technology have led to improved stability and dissolution profiles, particularly for rifampicin, a drug known for its variable absorption [23,24]. Earlier formulations raised concerns about reduced rifampicin bioavailability when combined with isoniazid; however, newer WHO-prequalified FDCs have demonstrated more consistent pharmacokinetic profiles [23,24,29].

Despite these advantages, certain pharmacological challenges remain [20,25,27]. Fixed ratios may not be optimal for all patients, particularly in cases of malnutrition, comorbidities, or drug–drug interactions, such as with antiretroviral therapy in HIV co-infected children [25,27,31]. In such scenarios, individualized dosing may be required to achieve therapeutic targets [25,27]. Nevertheless, for the majority of pediatric patients, FDCs provide a rational and effective approach that balances pharmacological efficacy with practical considerations [18,29].

## 4. Advantages of FDCs in Pediatric TB Treatment

Table 2 summarizes the advantages of FDCs in pediatric TB.

### 4.1. Improved Adherence

Adherence is a cornerstone of successful TB therapy, particularly in pediatric populations where treatment is prolonged and entirely dependent on caregivers [2,18,20]. FDCs substantially reduce pill burden by consolidating multiple drugs into a single formulation, simplifying daily administration [18,29]. This simplification decreases treatment fatigue and improves the likelihood that full regimens are completed [20,29]. Caregiver-related barriers—such as misunderstanding dosing schedules or difficulties managing multiple medications—are also mitigated [20]. Evidence from programmatic settings suggests that simplified regimens are associated with higher treatment completion rates and reduced loss to follow-up [18,20,28,29].

### 4.2. Reduction in Medication Errors

The use of separate drug formulations increases the risk of dosing inaccuracies, including incorrect dose calculations, missed doses, or omission of a drug component [19,20]. This risk is amplified in pediatric care due to weight-based dosing and the need for dose adjustments as children grow [25,26]. FDCs standardize drug ratios and reduce the number of individual dosing decisions required, thereby minimizing the potential for human error [18,29]. This is particularly relevant in low-resource settings where healthcare supervision may be limited [1,2,18].

### 4.3. Prevention of Drug Resistance

The emergence of drug-resistant TB is closely linked to inadequate or inconsistent therapy [6,7,29]. When drugs are administered separately, there is a risk that one or more components may be omitted, leading to functional monotherapy [29,30]. FDCs inherently prevent this scenario by ensuring that all essential drugs are taken together in the correct proportions [18,29]. This contributes to maintaining effective multidrug therapy throughout the treatment course, thereby reducing the risk of resistance development and supporting global TB control efforts [6,7,18,30].

### 4.4. Child-Friendly Formulations and Acceptability

Pediatric FDCs are specifically designed to address the unique needs of children [18,29]. Dispersible tablets that can be dissolved in small volumes of water facilitate administration in infants and young children who cannot swallow pills [18,29]. Improvements in taste masking and palatability are critical, as unpleasant taste has been identified as a major barrier to adherence [19,27]. The availability of flavored, easy-to-administer formulations enhances acceptability and reduces resistance to medication intake [20,29].

### 4.5. Simplification of Treatment Delivery and Health System Benefits

FDCs not only benefit patients and caregivers but also simplify treatment delivery at the health system level [18,29]. Standardized formulations facilitate procurement, storage, and distribution, reducing logistical complexity [18]. Healthcare providers benefit from simplified prescribing practices aligned with WHO weight-band dosing guidelines [18,26]. This standardization can improve consistency of care across different settings and reduce training requirements for healthcare workers [1,18].

### 4.6. Alignment with WHO Recommendations and Global Policy

The WHO strongly endorses the use of quality-assured pediatric FDCs as part of standard TB treatment protocols [3,18]. Their inclusion in international guidelines reflects robust evidence supporting their clinical and operational advantages [2,18]. The adoption of FDCs is also aligned with broader global health strategies aimed at improving treatment adherence, reducing drug resistance, and achieving TB elimination targets [1,18].

### 4.7. Potential Impact on Treatment Outcomes

Although randomized controlled trial data in pediatric populations are limited, available evidence suggests that FDCs contribute to improved treatment outcomes, including higher cure rates and lower default rates [27,28,29,31]. By addressing key barriers such as adherence, dosing errors, and formulation acceptability, FDCs play an important role in optimizing the overall effectiveness of TB therapy in children [18,20,29].

## 5. Limitations and Challenges

Despite the numerous advantages associated with FDCs, several limitations and challenges must be carefully considered when implementing their use in pediatric TB treatment (Table 3) [20,25,27]. These limitations are primarily related to pharmacological flexibility, safety monitoring, variability in drug exposure, and health system constraints [18,20,25]. Although pediatric FDCs represent an important advancement in the treatment of childhood TB, their benefits should be interpreted alongside relevant limitations. Their main strengths include simplification of multidrug therapy, reduction in pill burden, improved palatability through dispersible formulations, fewer medication errors, and better alignment with WHO-recommended weight-band dosing. These characteristics are particularly valuable in children, whose treatment depends heavily on caregivers and health-system support. However, FDCs also reduce the flexibility of individual drug dose adjustment, which may be problematic in children with malnutrition, rapid weight changes, drug toxicity, HIV co-infection, pharmacogenetic variability, or other comorbidities requiring tailored treatment. In particular, genetic differences affecting drug metabolism, such as *NAT2* acetylator status for isoniazid, may contribute to inter-individual variability in drug exposure and toxicity risk, but fixed drug ratios do not allow dose modification of single components according to individual pharmacogenetic profiles. In addition, although newer formulations have improved drug exposure, pharmacokinetic variability remains a concern, especially for rifampicin and in vulnerable pediatric subgroups. The attribution of adverse events to a specific drug is also more difficult when several agents are combined in a single formulation. Finally, the clinical benefits of FDCs may be limited by inconsistent access, supply-chain constraints, and variable implementation across settings. Therefore, while pediatric FDCs should be regarded as a cornerstone of simplified TB treatment, their use requires careful clinical monitoring, availability of alternative single-drug formulations when needed, and further pharmacokinetic, pharmacogenetic, and outcome studies in diverse pediatric populations.

### 5.1. Limited Dose Flexibility

One of the most significant limitations of FDCs is the inability to independently adjust the dose of individual drug components [18,25]. Pediatric TB management often requires dose modifications based on age, weight changes during treatment, comorbidities, or drug-related toxicities [25,26,27]. In situations such as hepatotoxicity, where specific drugs such as isoniazid or pyrazinamide may need to be reduced or temporarily discontinued, FDCs can complicate clinical management [19,20]. This lack of flexibility may necessitate switching back to separate formulations, potentially increasing treatment complexity and the risk of errors [20].

### 5.2. Pharmacokinetic Variability and Drug Exposure

Inter-individual variability in drug pharmacokinetics remains a concern, particularly in children [22,23,24,25,26,27]. Factors such as age, nutritional status, genetic polymorphisms, including acetylator status for isoniazid, and comorbid conditions can influence drug absorption and metabolism [22,25]. Rifampicin, in particular, has historically shown variable bioavailability in FDC formulations [23,24,29]. Although modern WHO-prequalified FDCs have improved pharmacokinetic performance, concerns persist regarding subtherapeutic drug concentrations in certain populations, including malnourished children and those co-infected with HIV [24,27,31].

### 5.3. Challenges in Managing Adverse Drug Reactions

When adverse drug reactions occur, identifying the causative agent can be more difficult with FDCs compared to single-drug formulations [19,20]. This is particularly relevant in pediatric patients, where monitoring and reporting of side effects may be less precise [20]. In cases of suspected drug toxicity, clinicians may need to discontinue the entire FDC and reintroduce individual drugs sequentially to identify the responsible compound [20]. This process can delay treatment and increase the risk of non-adherence [20].

### 5.4. Drug–Drug Interactions

Drug–drug interactions represent an additional challenge, especially in children receiving concomitant therapies [25,30,31]. Rifampicin is a potent inducer of cytochrome P450 enzymes and can significantly alter the pharmacokinetics of other medications, including antiretroviral drugs used in HIV-infected children [25,31]. The fixed ratios in FDCs limit the ability to adjust individual components to mitigate such interactions, potentially complicating co-treatment strategies [25,31].

### 5.5. Special Populations and Clinical Scenarios

Certain pediatric populations may require individualized therapeutic approaches that are not fully compatible with standard FDC formulations [25,27,31]. These include children with severe malnutrition, hepatic or renal impairment, and those with extrapulmonary or severe forms of TB such as TB meningitis [11,24,27,31]. In such cases, achieving optimal drug exposure may necessitate tailored dosing strategies that cannot be easily implemented with fixed-dose regimens [25,27].

### 5.6. Availability, Cost, and Supply Chain Constraints

Although FDCs are recommended by international guidelines, their availability remains inconsistent across different settings [3,18]. While access limitations are commonly reported in low- and middle-income countries, gaps in availability also exist in several high-income countries where tuberculosis is relatively uncommon [1,18]. In these non-endemic settings, limited market demand, regulatory barriers, and lack of commercial incentives may restrict the availability of pediatric FDC formulations [18,29]. This can create practical challenges in clinical management, often requiring the use of adult formulations that must be manipulated to achieve appropriate dosing [19,20]. Such practices may compromise dosing accuracy, increase the risk of medication errors, and negatively impact adherence [19,20,29]. Consequently, the lack of access to child-friendly FDCs represents a relevant issue not only in resource-limited settings but also in high-income countries, highlighting the need for broader global access and harmonization of regulatory and supply mechanisms [1,18,29].

### 5.7. Implementation and Health System Barriers

The successful integration of FDCs into national TB programs requires adequate training of healthcare providers, consistent supply systems, and effective monitoring frameworks [1,18]. In settings with limited healthcare infrastructure, challenges such as inadequate dosing supervision, poor record-keeping, and limited pharmacovigilance capacity may reduce the potential benefits of FDCs [1,18,20]. Additionally, variability in guideline implementation across regions can lead to inconsistent use of these formulations [18].

### 5.8. Evidence Gaps and Research Needs

While existing data support the use of FDCs in pediatric TB, important evidence gaps remain [27,29,31,32]. High-quality randomized controlled trials in children are limited, and long-term outcomes—including relapse rates and the impact on drug resistance—are not fully characterized [28,31,32]. Further research is needed to optimize drug ratios, evaluate pharmacokinetics in diverse pediatric subpopulations, and assess real-world effectiveness across different healthcare settings [25,27,31,32].

## 6. Clinical Evidence

Clinical evidence supporting the use of FDCs in pediatric TB derives from a combination of pharmacokinetic studies, observational cohorts, programmatic data, and a limited number of clinical trials (Table 4) [26,27,28,29,31].

While randomized controlled trials specifically designed for pediatric populations remain relatively scarce, the available body of evidence consistently supports the clinical utility of FDCs in improving treatment delivery and outcomes [27,29,31].

Pharmacokinetic (PK) studies have played a central role in validating pediatric FDC formulations [22,23,24,25,26]. Early concerns regarding suboptimal drug exposure, particularly for rifampicin, prompted revisions in WHO dosing recommendations and the development of improved formulations [3,18,23,24]. Subsequent PK studies have demonstrated that current WHO-recommended, weight-band-based pediatric FDCs generally achieve target plasma concentrations for first-line drugs in most children [16,19,20]. However, variability persists, especially among younger children, malnourished populations, and those co-infected with HIV, highlighting the importance of ongoing pharmacological monitoring and research [11,19,21].

Observational studies and programmatic data from high TB-burden countries provide important insights into real-world effectiveness [18,20,29]. Several national TB programs have reported improved treatment adherence and higher completion rates following the introduction of pediatric FDCs [18,20,29]. These improvements are largely attributed to reduced pill burden, simplified dosing schedules, and increased acceptability of child-friendly formulations [20,29]. In addition, caregiver satisfaction and ease of administration have been consistently reported as favorable outcomes associated with FDC use [20,29].

Evidence also suggests that FDCs may contribute to reducing loss to follow-up during treatment [18,20,29]. Simplified regimens facilitate directly observed therapy and community-based treatment approaches, which are commonly implemented in resource-limited settings [1,18]. By decreasing the complexity of drug administration, FDCs support more consistent treatment delivery and monitoring [18,20].

Comparative studies between FDCs and separate drug formulations in pediatric populations are limited but generally indicate comparable or improved outcomes with FDC use [27,29,31]. These include similar or higher rates of treatment success, defined by cure or treatment completion, and no significant increase in adverse events [29,31]. Importantly, the use of FDCs has not been associated with higher rates of treatment failure, supporting their safety and efficacy profile [29,31].

Safety data indicate that pediatric FDCs are generally well tolerated [29,31]. The incidence and type of adverse drug reactions appear consistent with those observed with individual drug formulations [19,29]. However, as noted previously, the attribution of specific adverse effects to individual components remains a challenge in FDC-based regimens [20,29].

Programmatic evaluations have also highlighted the role of FDCs in strengthening health systems [18,29]. Simplified procurement, storage, and distribution processes contribute to improved drug availability and reduced logistical burden [18]. Furthermore, standardized dosing aligned with WHO guidelines enhances consistency of care across different regions and healthcare levels [18,26].

Despite these positive findings, important evidence gaps remain [27,29,31,32]. High-quality randomized controlled trials specifically evaluating long-term outcomes, relapse rates, and resistance patterns in children treated with FDCs are still needed [28,31,32]. Additionally, more data are required to assess the performance of FDCs in special populations, including infants, children with severe malnutrition, and those receiving concomitant therapies such as antiretroviral treatment [24,25,27,31].

The available clinical evidence supporting pediatric FDCs should be interpreted with caution, as direct high-quality pediatric data remain limited. Although programmatic reports, observational studies, and pharmacokinetic analyses generally support the use of FDCs by showing improved acceptability, simplified administration, and adequate drug exposure in many children, randomized controlled trials specifically comparing pediatric FDCs with separate formulations are scarce. Moreover, much of the available evidence is derived from heterogeneous settings, includes relatively small pediatric subgroups, or focuses on pharmacokinetic rather than long-term clinical outcomes. Data on relapse, acquired drug resistance, adverse event attribution, and effectiveness in vulnerable populations, such as infants, malnourished children, children with HIV co-infection, and those with severe or extrapulmonary TB, remain insufficient. Therefore, while current evidence and WHO recommendations support the routine use of quality-assured pediatric FDCs, their benefits should not be overinterpreted as being fully established by robust comparative pediatric trials. Further prospective, adequately powered studies are needed to better define their impact on treatment success, safety, relapse rates, pharmacokinetic target attainment, and resistance prevention across diverse pediatric populations.

## 7. Future Research Directions: Nanotechnology-Based Approaches in Pediatric Tuberculosis

Emerging nanotechnology-based drug delivery systems represent a promising future direction for the treatment of pediatric TB, particularly because many of the current limitations of anti-TB therapy in children are related to poor palatability, variable absorption, prolonged treatment duration, high pill burden, and difficulty achieving reliable drug exposure in vulnerable populations [33,34,35,36]. Nanoformulations of first-line anti-TB drugs, including rifampicin, isoniazid, pyrazinamide, and ethambutol, have been investigated using several platforms such as polymeric nanoparticles, lipid-based nanocarriers, liposomes, nanosuspensions, nanocapsules, nanoemulsions, and inhalable particulate systems [34,35,36,37,38]. These systems may offer important pharmacokinetic advantages by improving drug solubility and stability, enhancing oral bioavailability, protecting drugs from premature degradation, and enabling sustained or controlled release [34,35,36,37]. For drugs such as rifampicin, which is characterized by variable absorption and bioavailability, nanocarrier-based delivery may theoretically improve systemic exposure and reduce fluctuations in plasma concentrations [34,35]. In addition, nanoparticle systems may allow co-encapsulation of multiple anti-TB agents, supporting synchronized multidrug delivery and potentially reducing the risk of functional monotherapy, a principle conceptually aligned with the rationale for FDCs [35,36].

From a pediatric perspective, nanotechnology-based approaches may be particularly relevant if they can be developed into child-friendly oral dispersible, taste-masked, or low-volume formulations [33]. Recent pediatric-focused discussions emphasize that nanotechnology could help overcome problems associated with manipulating adult tablets, inaccurate dose splitting, unpleasant taste, and subtherapeutic or toxic concentrations caused by developmental pharmacokinetic differences [33]. Beyond oral formulations, inhalable nanocarriers are also of interest because pulmonary delivery may increase drug concentrations at the primary site of infection, promote uptake by alveolar macrophages, reduce systemic exposure, and potentially limit dose-related toxicity [36,37,38]. Targeted delivery strategies, including surface modification with ligands such as mannose or hyaluronic acid, may further enhance macrophage uptake and localization within infected tissues [34]. These features could be especially valuable in children with severe disease, malnutrition, HIV co-infection, or extrapulmonary involvement, although pediatric-specific evidence remains very limited.

Despite these potential advantages, nanotechnology-based approaches remain largely experimental and should not yet be considered an alternative to currently recommended pediatric FDCs. Most available data derive from in vitro studies, formulation research, animal models, or adult-oriented preclinical work, while direct pediatric pharmacokinetic, safety, tolerability, and efficacy data are scarce [33,34,35,36,37,38]. Important translational challenges include ensuring reproducible large-scale manufacturing, long-term formulation stability, affordability, regulatory standardization, dose flexibility across pediatric weight bands, and compatibility with existing TB treatment programs [35,36]. Safety is also a critical concern, as nanoparticle size, surface charge, excipients, biodistribution, biodegradation, and accumulation in organs may influence toxicity, particularly in infants and young children with immature metabolic and elimination pathways [33,36,38]. Future studies should therefore prioritize pediatric-appropriate nanoformulations of first-line anti-TB drugs, comparative pharmacokinetic studies against WHO-recommended FDCs, evaluation in vulnerable pediatric subgroups, acceptability studies, and rigorous assessment of long-term safety and clinical outcomes. At present, nanotechnology should be viewed as a promising research frontier that may complement, rather than replace, optimized pediatric FDCs in future TB treatment strategies.

## 8. Conclusions

Pediatric FDCs represent an important tool in the treatment of childhood TB, particularly because they simplify multidrug therapy, reduce pill burden, improve ease of administration, and may help limit medication errors [18,20,29]. Their alignment with WHO-recommended weight-band dosing and the availability of dispersible, child-friendly formulations have addressed several practical barriers that historically complicated pediatric TB treatment [18,26,29].

However, the current evidence base should be interpreted with caution. Although pharmacokinetic studies, programmatic experience, and observational data generally support the use of pediatric FDCs, direct high-quality comparative pediatric trials remain limited [27,29,31,32]. Important uncertainties persist regarding long-term outcomes, relapse rates, acquired drug resistance, adverse event attribution, and effectiveness in vulnerable populations such as infants, malnourished children, children living with HIV, and those with severe or extrapulmonary TB [24,25,27,31,32].

Pharmacokinetic variability also remains a clinically relevant concern, particularly for rifampicin and isoniazid exposure, and fixed drug ratios may limit individualized dose adjustment in children with comorbidities, drug–drug interactions, toxicity, or pharmacogenetic differences [22,23,24,25,26,27,31]. In addition, access to quality-assured pediatric FDCs remains uneven across regions, limiting their potential impact in some settings [1,18,29].

Overall, pediatric FDCs should be considered a valuable and guideline-supported approach for simplifying TB treatment in children, rather than a definitive solution to all therapeutic challenges [18,29]. Further pediatric-specific pharmacokinetic, pharmacogenetic, clinical outcome, and implementation studies are needed to better define their optimal use, especially in vulnerable populations and diverse healthcare settings.

## Figures and Tables

**Table 1:** WHO-Recommended First-Line Anti-TB Drugs in Pediatric FDCs.

	Number of Tablets		
Weight (kg)	Intensive Phase HRZ 50/75/150 mg	E 100 mg *	Continuation Phase HR 50/75 mg
4–<8	1	1	1
8–<12	2	2	2
12–<16	3	3	3
16–<25	4	4	4
≥25	Adult dosages recommended		

H, isoniazid; R, rifampicin; Z, pyrazinamide; E, ethambutol. * To administer in case of high prevalence of HIV infection or resistance to isoniazid.

**Table 2:** Advantages of FDCs in Pediatric TB.

Domain	Advantage	Impact
Adherence	Reduced pill burden	Higher completion rates
Safety	Fewer dosing errors	Improved safety
Resistance	Ensures multidrug intake	Reduced resistance
Acceptability	Child-friendly forms	Better compliance
Systems	Simplified logistics	Easier implementation

**Table 3:** Limitations and Challenges of FDCs in Pediatric TB.

Category	Limitation	Implication
Dosing	Fixed ratios	Limited flexibility
PK	Variable exposure	Subtherapeutic risk
Safety	Hard ADR attribution	Complex management
Interactions	Limited adjustment	HIV co-treatment issues
Access	Variable availability	Inequity

PK, pharmacokinetics; ADR, adverse drug reaction.

**Table 4:** Summary of Clinical Evidence on Pediatric FDCs.

Evidence Type	Findings	Limitations
PK studies	Adequate exposure	Variability
Observational	Better adherence	Confounding
Programmatic	Improved delivery	Reporting limits
Comparative	Similar/better outcomes	Few RCTs

PK, pharmacokinetic; RCTs, randomized controlled trials.

## Data Availability

No new data were created or analyzed in this study.
